# Systemic inflammatory response index in the differentiation of unstable angina pectoris and non-ST elevation myocardial infarction

**DOI:** 10.1097/MD.0000000000046726

**Published:** 2025-12-12

**Authors:** Serdar Akyel, Abdulkadir Yildiz

**Affiliations:** a Department of Cardiology, Kastamonu University School of Medicine, Kastamonu, Turkey.

**Keywords:** acute coronary syndrome, inflammation, NSTEMI, SIRI, USAP

## Abstract

The systemic inflammatory response index (SIRI), calculated from routine complete blood count parameters, has emerged as a potential marker of inflammation and a predictor of prognosis in various cardiovascular conditions. This study aimed to evaluate the utility of SIRI in differentiating non-ST-elevation myocardial infarction (NSTEMI) from unstable angina pectoris (USAP) in patients presenting with acute chest pain. In this retrospective observational study, patients admitted to the emergency department with chest pain and subsequently diagnosed with USAP or NSTEMI were included. Only patients with negative high-sensitive troponin-I levels at the time of admission were enrolled in both groups. During follow-up, those who showed an increase in troponin levels were classified as NSTEMI, whereas patients without troponin elevation were classified as USAP. The SIRI was calculated using blood samples obtained at the time of admission to the emergency department by multiplying the neutrophil and monocyte counts and dividing by the lymphocyte count. Troponin positivity was used to distinguish NSTEMI from USAP. SIRI was assessed at admission in patients with chest pain and normal troponin. Higher SIRI levels in NSTEMI suggest its potential for early differentiation from USAP. Multivariate logistic regression analysis was performed to determine independent predictors of troponin positivity, and receiver operating characteristic curve analysis was used to assess diagnostic performance. A total of 151 patients were included in the analysis. Admission SIRI values were significantly higher in the troponin-positive (NSTEMI) group compared with the troponin-negative (USAP) group (*P* < .001). In multivariate analysis, higher admission SIRI levels independently predicted troponin positivity (odds ratio [OR], 1.47; 95% confidence interval [CI], 1.17–1.84; *P* < .001). Receiver operating characteristic analysis revealed an area under the curve of 0.71 (95% CI 0.63–0.79, *P* < .001). A cutoff value of 1.58 for SIRI demonstrated 72% sensitivity and 69% specificity in predicting troponin positivity. SIRI, a readily available, inexpensive, and rapidly obtainable index derived from standard complete blood count parameters, may serve as a valuable adjunctive tool for the early differentiation of NSTEMI from USAP in patients with acute chest pain. Its ease of calculation within minutes of admission makes it suitable in diverse healthcare settings.

## 1. Introduction

Acute coronary syndromes (ACS) encompass a spectrum of clinical entities resulting from acute impairment of coronary blood flow, ultimately leading to myocardial injury. ACS remains a primary global health concern due to its substantial contribution to morbidity and mortality.^[1]^ According to the 2023 American Heart Association statistics, 1.266,000 patients in the United States were discharged with a diagnosis of ACS in 2019.^[2]^ Of these, 1.248,000 had myocardial infarction as the sole diagnosis, whereas 18,000 were diagnosed with unstable angina. Overall, ACS accounts for approximately half of all cardiovascular-related deaths worldwide.^[3]^

Among ACS presentations, unstable angina pectoris (UAP) and non-ST-elevation myocardial infarction (NSTEMI) together constitute approximately 80% of cases, with the remainder represented by ST-elevation myocardial infarction (STEMI). STEMI is associated with the highest mortality, primarily due to severe ischemia secondary to complete coronary artery occlusion. Although the in-hospital mortality rates of UAP and NSTEMI are lower compared with STEMI, long-term prognosis may still be adversely affected, particularly due to the risk of recurrent myocardial infarction after hospital discharge.^[4]^

Early STEMI diagnosis is often feasible based on characteristic changes observed in the electrocardiogram (ECG) obtained at the time of presentation to the emergency department. In contrast, in the early stages of the disease, differentiation between UAP and NSTEMI is not possible solely based on ECG findings or elevated cardiac biomarkers. According to the 4th Universal Definition of Myocardial Infarction, the diagnosis of NSTEMI relies on a rise and/or fall of cardiac troponin values above the 99th percentile upper reference limit, accompanied by evidence of myocardial ischemia. In contrast, patients with acute chest pain and no troponin elevation but with clinical features of ischemia are classified as having UAP. In our study, both patient groups were initially evaluated with highly sensitive troponin-I at admission. Only those with negative troponin levels were included, and subsequent troponin elevation during follow-up determined the classification as NSTEMI, while patients without elevation were classified as UAP. This approach underscores the pivotal role of troponin measurement in early differentiation and risk stratification in acute coronary syndromes. This diagnostic challenge has prompted the search for a readily available, rapid, and highly sensitive marker capable of distinguishing UAP from NSTEMI during emergency admission. In recent years, numerous studies have investigated various hematological parameters derived from routine complete blood count analysis as potential candidates for this purpose.^[5–8]^

Leukocytes play a pivotal role in the pathogenesis of thrombosis and the development of atherosclerotic plaques. Numerous studies have demonstrated that total leukocyte count and its subtypes exhibit significant variations in response to the pathophysiological processes underlying atheromatous plaque formation and in a wide range of cardiovascular disorders.^[9–13]^ Among leukocyte subtypes, neutrophil and monocyte counts tend to increase in the presence of inflammation, ischemia, and oxidative stress.^[14,15]^ In contrast, lymphocyte counts are often reduced under systemic inflammation and physiological stress.^[16]^ Based on these observations, composite inflammatory indices have been proposed better to reflect the balance between pro-inflammatory and regulatory immune responses. One such marker, the systemic inflammatory response index (SIRI), is calculated as: SIRI = Neutrophil count × Monocyte count/Lymphocyte. Emerging evidence suggests that SIRI is closely associated with the progression of coronary atherosclerosis and with adverse outcomes in acute myocardial infarction.^[8]^

Given that NSTEMI and UAP are both clinical manifestations within the spectrum of acute coronary syndrome, elevations in systemic inflammatory markers are expected in both conditions. However, the magnitude of these inflammatory responses is likely greater in NSTEMI than in UAP, reflecting the more extensive myocardial injury and inflammatory activation associated with myocardial necrosis. This differential inflammatory burden may provide a valuable basis for early distinction between the two entities at the time of initial hospital admission.

Accordingly, the present study aimed to evaluate the diagnostic utility of the SIRI in differentiating NSTEMI from UAP in patients presenting with a preliminary diagnosis of acute coronary syndrome.

## 2. Materials and methods

### 2.1. Study design and setting

This retrospective observational study was conducted at the Cardiology Clinic of Kastamonu Education and Research Hospital between June 2023 and January 2024.

### 2.2. Ethical considerations

The study used the ethical principles outlined in the Declaration of Helsinki (revised in 2013, Brazil). The Ethics Committee and Research Committee of Kastamonu Education and Research Hospital approved it.

### 2.3. Study population

The study included patients who presented to the emergency department with chest pain, had non-elevated initial high-sensitive troponin-I levels, and were subsequently diagnosed with UAP or NSTEMI based on further clinical, electrocardiographic, and laboratory evaluations. In accordance with European Society of Cardiology guidelines, highly sensitive troponin-I levels were measured at 0, 1, and 3 hours after admission. The initial troponin level was obtained at presentation, and patients who demonstrated an increase at the 1st or 3rd hour were classified as NSTEMI. In contrast, those without troponin elevation were classified as UAP. Patients of all ethnic backgrounds and both sexes were eligible for inclusion.

### 2.4. Exclusion criteria

Patients were excluded if they had a known history of malignancy, acute or chronic infection, septicemia, collagen vascular disease, hematologic disorders, acute or chronic hepatic or renal failure, or were receiving immunosuppressive therapy. Additional exclusion criteria included a body temperature ≥ 38°C and a white blood cell count > 12,000/μL or < 4000/μL at presentation.

### 2.5. Data collection and definitions

The eligible patients’ demographic characteristics and clinical data, including history of coronary artery disease, previous cardiac surgery, smoking status, and medication use, as well as laboratory results, were retrieved from the hospital’s electronic medical record system. Troponin levels were assessed at 0, 1, and 3 hours after admission, in line with European Society of Cardiology guidelines. Troponin elevation was defined as values exceeding the 99th percentile upper reference limit (16 ng/L) for high-sensitive troponin-I. Patients with elevated troponin levels during this period and without ST-segment elevation on the admission ECG were classified as NSTEMI. Patients without troponin elevation during hospitalization and without ischemic ECG changes – defined as new or dynamic ST-segment depression (≥0.5 mm), transient ST-segment elevation (≥0.5 mm), or T-wave inversion (≥1 mm) in two or more contiguous leads – were classified as having UAP.

### 2.6. Laboratory parameters

Upon admission, all patients underwent complete blood count (CBC), serum biochemistry, lipid profile, coagulation profile, and cardiac biomarker testing, with results recorded in the hospital’s electronic medical record system. Blood samples were analyzed in the same laboratory using standardized protocols and identical reagent kits to ensure consistency.

Red blood cell and platelet counts were determined using the impedance (resistance) method, whereas leukocyte counts were measured by optical laser scattering. All hematological parameters were obtained using a Sysmex XE-2100 hematology analyzer (Roche Diagnostics Corp., Indianapolis).

#### 2.6.1. SIRI calculation and troponin measurement

High-sensitivity troponin I levels were quantified with a UniCel DxI 600 immunoassay system (Beckman Coulter, Brea). A value exceeding the 99th percentile upper reference limit (16 ng/L) was considered elevated. The systemic inflammatory response index (SIRI) was calculated for each patient as follows: SIRI = Neutrophil count × Monocyte count/Lymphocyte count. Standard 12-lead ECGs were obtained for all patients at presentation using a GE MAC 2000 ECG system (GE Healthcare, Chicago).

### 2.7. Statistical analysis

For statistical evaluations, the Statistical Package for the Social Sciences (SPSS) (IBM SPSS Inc., Chicago) for Windows 20 and MedCalc 11.4.2 (MedCalc Software, Mariakerke, Belgium) software packages were used. The normality of the data distribution was assessed using the Kolmogorov–Smirnov test. Values with a normal distribution were presented as mean ± standard deviation, and values not normally distributed were presented as median. Categorical variables were presented as numbers and percentages. Differences between groups were analyzed using the independent sample *t* test for normally distributed parameters and the Mann–Whitney *U* test for non-normally distributed parameters. Predictive values of SIRI levels for NSTEMI were determined using receiver operating characteristic curve analysis, and the optimal cutoff point was determined using the Youden index. A *P* value of <.05 was considered statistically significant for all analyses. Variables with a *P* value <.1 in univariate analysis were included in the multivariate logistic regression analysis, and results were presented as odds ratios (OR) with 95% confidence intervals (CI).

## 3. Results

A total of 151 patients with non-elevated initial troponin levels were included in the analysis. The mean age of the study population was 63.5 ± 14.0 years, with 74% of participants being male. Patients were stratified into two groups according to the presence or absence of troponin elevation during follow-up. The groups baseline demographic, biochemical, and hematological characteristics are presented in Table 1. The two groups were comparable in terms of cardiovascular risk factors, history of coronary artery disease, and previous percutaneous coronary intervention or coronary artery bypass grafting.

**Table 1:** Baseline characteristics of the study population according to the development of troponin positivity during follow-up.

	Troponin (−) (n = 79)	Troponin (+) (n = 72)	*P* value
Age	64 ± 14	63 ± 14	.510
Hemoglobin	13.5 ± 1.7	13.6 ± 2.4	.808
Admission troponin	9 (7–12)	11 (6–13)	.510
Control troponin	12 (9–14)	250 (84–485)	<.001
RDW	15.4 ± 1.4	14 ± 1.6	.405
MPV	10 ± 1.4	9.9 ± 1.3	.693
PLT	212 ± 60	225 ± 65	.218
WBC	8.0 ± 2.2	10.2 ± 3.3	<.001*
Neutrophil	5.2 ± 2.1	7.2 ± 2.7	<.001*
Lymphocyte	2.1 ± 0.7	2.1 ± 1.1	.793
Monocyte	0.6 ± 0.2	0.7 ± 0.3	.041*
SIRI	1.42 (0.81–2.19)	3.59 (1.71–5.92)	<.001*
Total cholesterol	175 ± 45	180 ± 41	.533
LDL	103 ± 35	114 ± 37	.078
HDL	41 ± 12	38 ± 11	.180
Triglycerides	115 (83–207)	94.5 (57–136)	.714
Uric acid	5.41 ± 1.23	5.65 ± 1.57	.505
Smoking	31 (42)	31 (46)	.657
DM	21 (27)	12 (17)	.141
HT	36 (46)	32 (44)	.890
Male gender	58 (73)	54 (75)	.824
EF	57 (40–60)	55 (41–60)	.005*
CAD	33 (42)	23 (32)	.212
CABG	11 (14)	9 (13)	.797
PCI	13 (17)	11 (15)	.843
Beta bloker	29 (38)	18 (27)	.169
ACE/ARB	32 (42)	25 (38)	.608
Statin	10 (13.2)	5 (7.6)	.280
ASA	33 (43)	18 (27)	.045*

Categorical variables were expressed as numbers and percentages; numerical variables were expressed as standard deviation or median (min–max).

ACE = angiotensin converting enzyme, ARB = angiotensin receptor blocker, ASA = acetyl salicylic acid, CABG = coronary artery by-pass graft, CAD = coronary artery disease, CI = confidence interval, DM = diabetes mellitus, EF = ejection fraction, FFR = fractional flow reserve, HDL = high-density lipoprotein, HT = hypertension, LDL = low-density lipoprotein, MPV = mean platelet volume, PCI = percutaneous coronary intervention, PCT = procalcitonin, PDW = platelet cell distirubition width, PLT = platelet count, RDW = red cell distribution width, SIRI = Systemic Inflammatory Response Index, WBC = white blood cells.

* *P* < .05 was considered statistically significant.

Compared with the troponin-negative group, patients in the troponin-positive group exhibited significantly higher white blood cell (WBC) counts, neutrophil counts, platelet counts, and monocyte counts, as well as lower low-density lipoprotein (LDL) cholesterol levels. In contrast, triglyceride levels were significantly lower in the troponin-positive group. The median SIRI value was also markedly higher in the troponin-positive group (*P* < .001, Table 1).

In the multivariate logistic regression analysis, all variables included in the model were evaluated simultaneously. SIRI was identified as a significant independent predictor of the outcome (OR 1.44, 95% CI 1.154–1.788, *P* = .002). ASA use also showed an independent association with the outcome (OR 2.586, 95% CI 1.149–5.817, *P* = .022). In contrast, LDL and monocyte count did not demonstrate statistically significant associations in the multivariate model (LDL: OR 1.006, 95% CI 0.995–1.016, *P* = .288; Monocyte: OR 1.001, 95% CI 0.999–1.002, *P* = .245). The overall Nagelkerke *R*^2^ for the multivariate model was 0.456, indicating a moderate explanatory power of the included variables. These findings suggest that, among the variables studied, SIRI and ASA use contribute most strongly to the outcome in this cohort (Table 2).

**Table 2:** Multivariate logistic regression analysis and univariate analysis to assess predictors of troponin elevation.

Variable	Univariate OR (95% CI)	*P* value	Multivariate OR (95% CI)	*P* value	Nagelkerke *R*^2^
SIRI	1.001 (0.990–1.002)	.002	1.44 (1.154–1.788)	.002	.28
LDL	1.008 (0.998–1.017)	.094	1.006 (0.995–1.016)	.288	.15
ASA use	2.152 (1.073–4.317)	.031	2.586 (1.149–5.817)	.022	.18
Monocyte	1.001 (1.000–1.002)	.041	1.001 (0.999–1.002)	.245	.12
Multivariate model (overall)	–	–	–	–	.456

Categorical variables were expressed as numbers and percentages; numerical variables were expressed as standard deviation or median (min–max).

Nagelkerke *R*^2^ values ranged from 0.12 to 0.28 for univariate models, and were 0.456 for the overall multivariate model.

ASA = acetylsalicylic acid, CI = confidence interval, LDL = low-density lipoprotein, OR = odds ratio, SIRI = Systemic Inflammatory Response Index.

* *P* < .05 was considered statistically significant.

**Figure 1.**
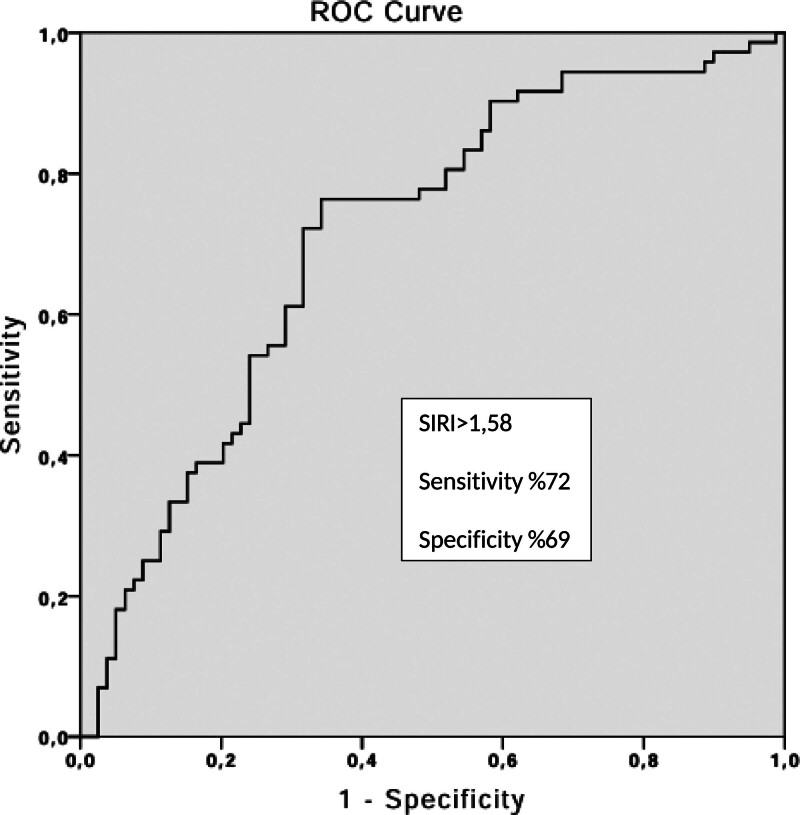
The receiver–operating characteristic curve analysis of Systemic Inflammatory Response Index (SIRI) for predicting troponin positivity. ROC = receiver operating characteristic.

Receiver operating characteristic curve analysis yielded an area under the curve of 0.71 (95% CI 0.63–0.79, *P* < .001). A SIRI threshold 1.58 predicted troponin positivity with 72% sensitivity and 69% specificity (Fig. 1).

## 4. Discussion

The present study demonstrated that elevated systemic inflammatory response index (SIRI) values measured at emergency department admission are associated with subsequent troponin elevation in patients presenting with acute chest pain. This finding highlights the potential link between systemic inflammation and acute myocardial injury. Notably, SIRI levels were significantly higher in patients diagnosed with NSTEMI compared with those with unstable angina pectoris (UAP), even before biochemical evidence of myocardial necrosis was present. These results suggest that SIRI may serve as an early indicator of inflammatory activation that precedes troponin release, thus offering additional diagnostic value in differentiating NSTEMI from UAP during the early phase of presentation.

ACS encompass a spectrum of clinical conditions characterized by acute myocardial ischemia and inflammation-driven plaque instability. While ECG findings provide immediate diagnostic information in ST-elevation myocardial infarction (STEMI), identifying non-ST-elevation events remains challenging due to the often subtle or absent early troponin changes. In this context, inflammatory biomarkers reflecting the immune response to endothelial injury and ischemia may complement cardiac enzymes in improving diagnostic precision.

Inflammation plays a pivotal role in the initiation, progression, and destabilization of atherosclerotic plaques.^[17–19]^ Oxidative stress and inflammatory activation trigger endothelial dysfunction and leukocyte recruitment, initiating a cascade that culminates in plaque rupture and myocardial injury. Elevated white blood cell (WBC) counts and increased levels of C-reactive protein have long been recognized as markers of heightened cardiovascular risk and plaque vulnerability.^[20–23]^ The interplay between inflammation and myocardial ischemia is therefore central to the pathophysiology of acute coronary syndromes.^[24]^

Leukocytes and their subtypes – particularly neutrophils, lymphocytes, and monocytes – play distinct roles in the inflammatory response underlying myocardial injury.^[25]^ Monocytes, through the release of cytokines and differentiation into macrophages, contribute to both plaque formation and post-ischemic inflammation.^[26,27]^ Elevated monocyte counts have been identified as predictors of adverse cardiovascular outcomes.^[28,29]^ Neutrophils exacerbate ischemic damage through oxidative stress and endothelial injury,^[30,31]^ whereas lymphopenia has been associated with poorer outcomes in ACS.^[32–35]^ Platelets also play a dual role in atherosclerosis. Their adhesion to the vascular endothelium facilitates plaque formation,^[36]^ whereas platelet activation promotes vascular inflammation and thrombus development.^[37]^ These cellular interactions provide the biological foundation for composite hematologic indices such as SIRI, which integrates neutrophil, monocyte, and lymphocyte counts to reflect the balance between inflammation and immune regulation.

Recent evidence supports the prognostic and diagnostic significance of systemic inflammatory indices in coronary artery disease. The systemic immune-inflammation index (SII) and SIRI have both been correlated with the extent and severity of coronary atherosclerosis, as reflected by angiographic scores such as the Gensini and SYNTAX indices.^[38,39]^ Moreover, SII has been linked to physiologically significant coronary stenosis assessed by fractional flow reserve.^[40]^ However, SIRI may have greater biological relevance, as it incorporates the monocyte component – one of the key mediators of atherosclerotic inflammation and myocardial injury. Previous studies have demonstrated that SIRI and SII levels are significantly higher in patients with acute coronary syndromes compared with those with stable coronary disease,^[8]^ and SIRI has shown stronger associations with major adverse cardiovascular events and long-term outcomes.^[41–43]^

The present study extends this evidence by demonstrating that systemic inflammation, as quantified by SIRI, precedes and predicts troponin elevation in patients with acute chest pain. This temporal relationship underscores the potential causal link between inflammatory activation and myocardial injury. SIRI may therefore represent a useful adjunctive biomarker to troponin, especially in the early differentiation of NSTEMI from UAP, when conventional diagnostic markers remain equivocal.

Several limitations should be acknowledged. The retrospective design and relatively small cohort size limit the generalizability of the findings. Additionally, other inflammatory markers such as C-reactive protein, cytokines, or serum amyloid A were not assessed, which could have strengthened the interpretation of the inflammatory–ischemic relationship. Finally, longitudinal evaluation of SIRI after discharge was not available, preventing assessment of its prognostic value over time.

## 5. Conclusion

Despite these limitations, our findings suggest that SIRI, as a novel and easily obtainable inflammatory biomarker, may be a valuable tool for early differentiation between NSTEMI and UAP, potentially assisting in guiding timely therapeutic decision-making. Nonetheless, further large-scale, prospective, and methodologically robust studies are warranted to validate these results and to clarify the clinical utility of SIRI in both diagnostic and prognostic contexts.

## Author contributions

**Conceptualization:** Serdar Akyel, Abdulkadir Yildiz.

**Data curation:** Serdar Akyel, Abdulkadir Yildiz.

**Formal analysis:** Serdar Akyel, Abdulkadir Yildiz.

**Funding acquisition:** Serdar Akyel, Abdulkadir Yildiz.

**Investigation:** Serdar Akyel, Abdulkadir Yildiz.

**Methodology:** Serdar Akyel, Abdulkadir Yildiz.

**Project administration:** Serdar Akyel, Abdulkadir Yildiz.

**Resources:** Serdar Akyel, Abdulkadir Yildiz.

**Software:** Serdar Akyel, Abdulkadir Yildiz.

**Supervision:** Serdar Akyel, Abdulkadir Yildiz.

**Validation:** Serdar Akyel, Abdulkadir Yildiz.

**Visualization:** Serdar Akyel, Abdulkadir Yildiz.

**Writing – original draft:** Serdar Akyel, Abdulkadir Yildiz.

**Writing – review & editing:** Serdar Akyel, Abdulkadir Yildiz.
